# Modifying the multi-arm multi-stage (MAMS) design for use in a phase II tuberculosis trial in sub-Saharan Africa with a time-to-event primary outcome

**DOI:** 10.1186/1745-6215-14-S1-P26

**Published:** 2013-11-29

**Authors:** Patrick Phillips, Michael Hoelscher, Daniel Bratton, Sunita Rehal, Norbert Heinrich, Georgette Plemper van Balen, Andrew Nunn, Rob Aarnoutse, Sonja Henne, Stephen Gillespie, Martin Boeree

**Affiliations:** 1 Radboud University Nijmegen Medical Centre, Nijmegen, The Netherlands; 2 Medical Centre of the University of Munich (LMU), Munich, Germany; 3 University of St Andrews School of Medicine, St. Andrews, UK; 4 MRC Clinical Trials Unit, London, UK

## Background

Tuberculosis (TB) causes significant morbidity and mortality globally. High efficacy in clinical trials of the current 4-drug 6-month regimen does not always translate into practice due to a heavy pill burden and long duration. New drugs are in late-phase clinical development, so trials that evaluate novel and existing drugs in combination are urgently needed. A multi-arm multi-stage (MAMS) design, with planned interim analyses allowing stopping of arms not showing benefit, has been developed and used in oncology. We have adapted this approach to create the PanACEA MAMS-TB 5-arm 3-stage phase II trial evaluating new treatments for TB.

## Methods

The transition from an endpoint of progression-free survival to one of negative culture conversion required several modifications including allowing for fixed follow-up of 12 weeks and targeting an increase in the hazard of the event rather than a decrease. The Weibull distribution was used in place of the exponential distribution to model a more appropriate increasing hazard. The option of a second interim analysis protects against slow recruitment.

## Results

In this paper, the modifications to the design will be explained with discussion around pair-wise and family-wise type I error rates, power and expected sample size in comparison with a fixed sample design.

## Conclusions

Reductions in expected sample size are moderate in this relatively small 5-arm study due to the absence of a real-time biomarker. Nevertheless, there is potential for faster evaluation of more regimens in a larger phase II/III design once better biomarkers are validated.

